# Crystal structure, Hirshfeld surface analysis and DFT studies of 4-methyl-2-({[4-(tri­fluoro­meth­yl)phen­yl]imino}­meth­yl)phenol

**DOI:** 10.1107/S2056989020009615

**Published:** 2020-07-21

**Authors:** Md. Serajul Haque Faizi, Emine Berrin Cinar, Onur Erman Dogan, Alev Sema Aydin, Erbil Agar, Necmi Dege, Ashraf Mashrai

**Affiliations:** a Department of Chemistry, Langat Singh College, B.R.A. Bihar University, Muzaffarpur, Bihar-842001, India; b Ondokuz Mayıs University, Faculty of Arts and Sciences, Department of Physics, Samsun, Turkey; c Ondokuz Mayıs University, Faculty of Arts and Sciences, Department of Chemistry, Samsun, Turkey; d Department of Pharmacy, University of Science and Technology, Ibb Branch, Ibb, Yemen

**Keywords:** crystal structure, 2-hy­droxy-5-methyl-benzaldehyde, 4-tri­fluoro­methyl-phenyl­amine

## Abstract

The title compound, C_15_H_12_F_3_NO, crystallizes with one mol­ecule in the asymmetric unit. The configuration of the C=N bond is *E* and there is an intra­molecular O—H⋯N hydrogen bond present, forming an *S*(6) ring motif. The dihedral angle between the mean planes of the phenol and the 4-tri­fluoro­methyl­phenyl rings is 44.77 (3)°. In the crystal, mol­ecules are linked by C—H⋯O inter­actions, forming polymeric chains extending along the *a*-axis direction. The Hirshfeld surface analysis indicates that the most important contributions to the crystal packing are from C⋯H/H⋯C (29.2%), H⋯H (28.6%), F⋯H/H⋯F (25.6%), O⋯H/H⋯O (5.7%) and F⋯F (4.6%) inter­actions. The density functional theory (DFT) optimized structure at the B3LYP/6-311 G(d,p) level is compared with the experimentally determined mol­ecular structure in the solid state. The HOMO–LUMO behaviour was elucidated to determine the energy gap. The crystal studied was refined as an inversion twin.

## Abstract 2

The title compound, C_15_H_12_F_3_NO, crystallizes with a single mol­ecule in the asymmetric unit. The phenol ring makes a dihedral angle of 44.77 (3)° with the benzene ring of the tri­fluoro­methyl group. In the crystal, mol­ecules are linked by C—H⋯O inter­actions, forming polymeric chain along the *b-*axis direction.

## Chemical context   

Over the past 25 years, there has been extensive research on the synthesis and use of Schiff base compounds in organic and inorganic chemistry as they have important medicinal and pharmaceutical applications. These compounds show biological activities including anti­bacterial, anti­fungal, anti­cancer and herbicidal activities (Desai *et al.*, 2001 ▸; Singh & Dash, 1988 ▸; Karia & Parsania, 1999 ▸). Schiff bases are also becoming increasingly important in the dye and plastics industries, as well as in liquid-crystal technology and for the mechanistic investigation of drugs used in pharmacology, biochemistry and physiology (Sheikhshoaie & Sharif, 2006 ▸). The present work is a part of an ongoing structural study of Schiff bases and their use in the synthesis of new organic, excited-state proton-transfer compounds and fluorescent chemosensors (Faizi *et al.*, 2016 ▸, 2018 ▸; Kumar *et al.*, 2018 ▸; Mukherjee *et al.*, 2018 ▸). We report here on the synthesis and crystal structure as well as the Hirshfeld surface analysis of the new compound, (I).
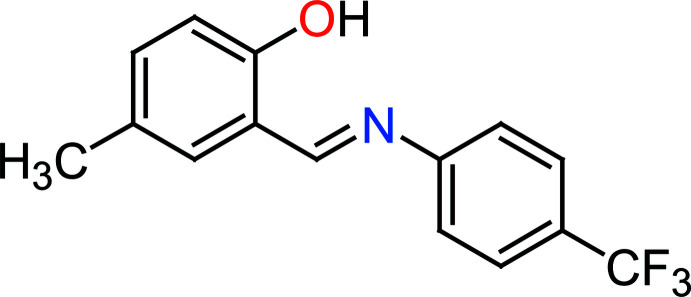


The results of calculations by density functional theory (DFT) carried out at the B3LYP/6-311 G(d,p) level are compared with the experimentally determined mol­ecular structure of (I) in the solid state.

## Structural commentary   

**Figure 1:**
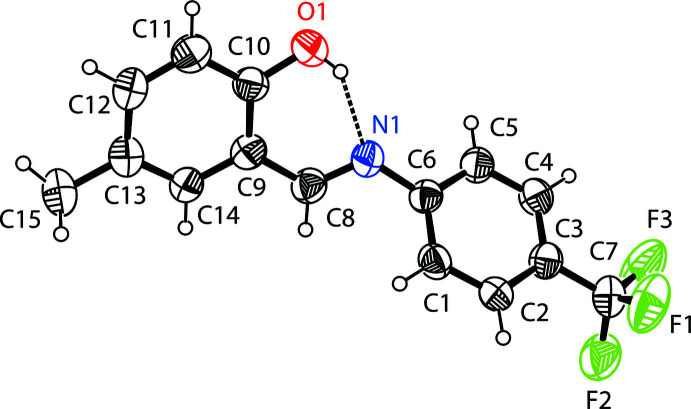
The mol­ecular structure of (I) with the atom-numbering scheme. Displacement ellipsoids are drawn at the 40% probability level. The intra­molecular O—H⋯N hydrogen bond (Table 1 ▸) is shown as a dashed line.

The mol­ecular structure of the title compound, (I), is illustrated in Fig. 1 ▸. There is an intra­molecular O—H⋯N hydrogen bond present (Table 1 ▸ and Fig. 1 ▸), forming an *S*(6) ring motif; this is a common feature in related imine–phenol compounds. The imine group displays a C9—C8—N1—C6 torsion angle of 170.1 (4)° while the mean plane of the phenol ring (C9–C14) is inclined to that of the 4-tri­fluoro­methyl­phenyl group (C1–C6) by 44.77 (3)°. The configuration of the C8=N1 bond is *E*. The C10—O1 bond length [1.357 (8) Å (experimental) and 1.342 Å (calculated)] indicates single-bond character (Ozeryanskii *et al.*, 2006 ▸), while the imine C8=N1 bond length [1.283 (8) Å (experimental) and 1.290 Å (calculated)] indicates double-bond character. All these data support the existence of the phenol–imine tautomer for (I) in its crystalline state. These features are similar to those observed in related 4-di­methyl­amino-*N*-salicylideneanilines (Pizzala *et al.*, 2000 ▸).

## Supra­molecular features   

**Figure 2:**
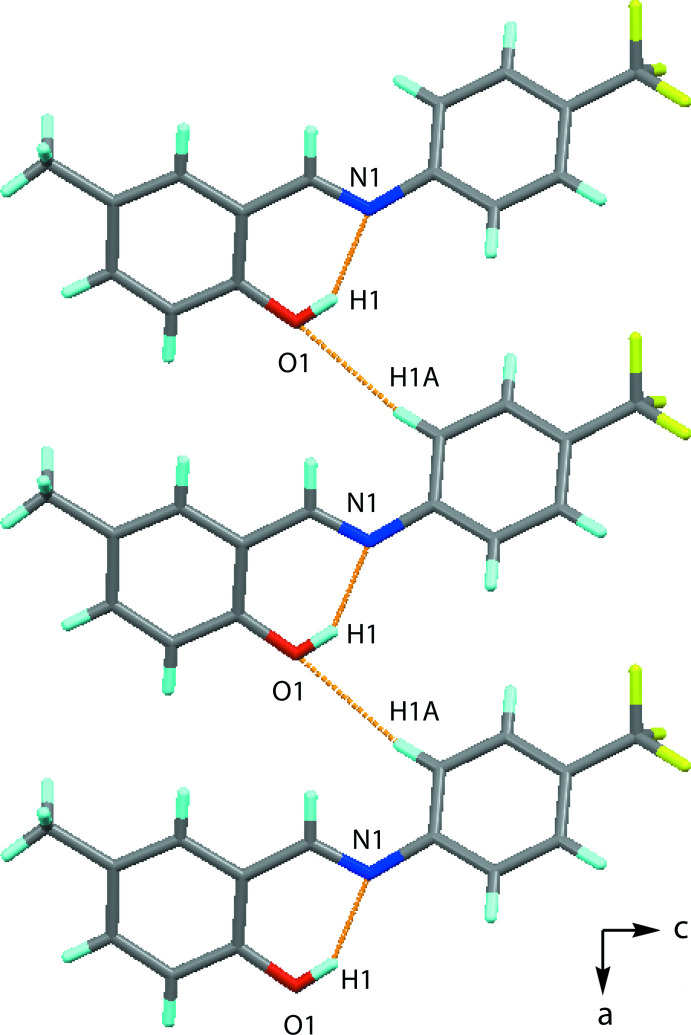
A view along the *b* axis of the polymeric chain formed *via* C—H⋯O inter­actions (see Table 1 ▸ for details).

**Figure 3:**
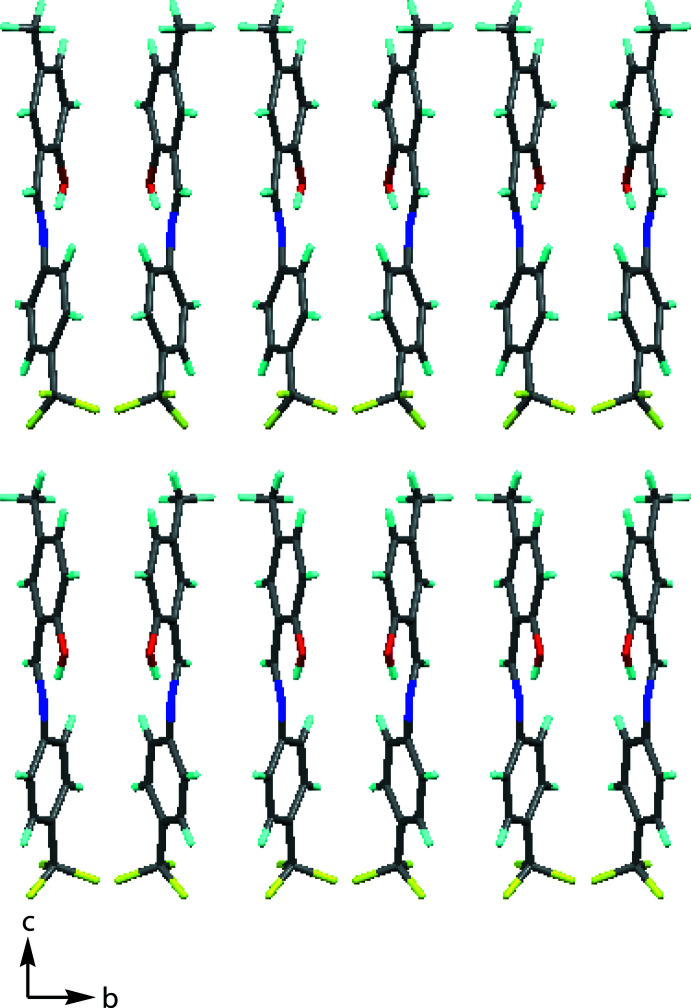
A view of the crystal packing along the *a* axis.

In the crystal of (I), mol­ecules are linked by inter­molecular C—H⋯O inter­actions, forming chains extending along the *a*-axis direction (Fig. 2 ▸ and Table 1 ▸). The crystal packing along the *a*-axis direction is shown in Fig. 3 ▸.

## Hirshfeld surface analysis and two-dimensional fingerprint plots   

**Figure 4:**
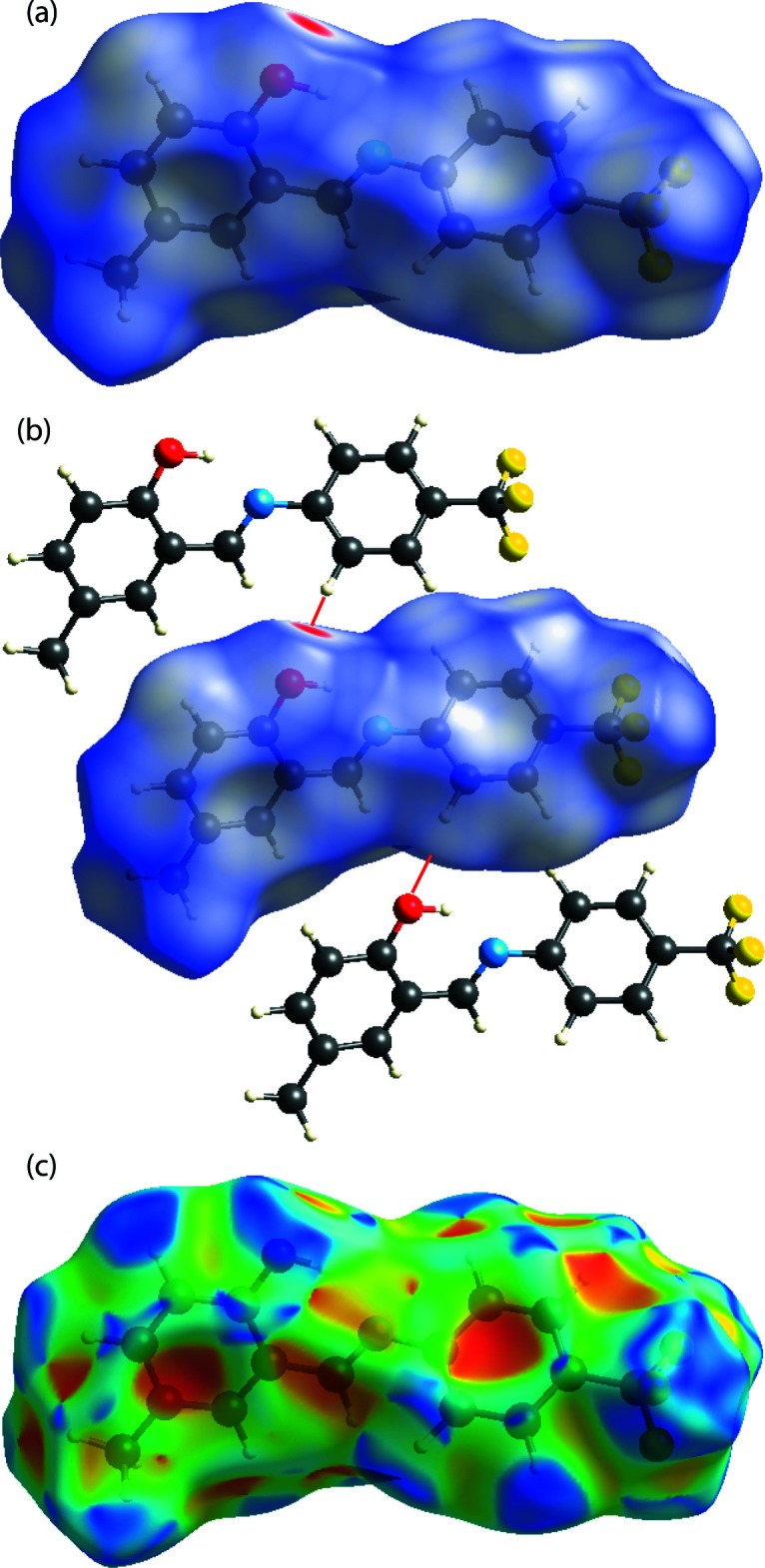
A view of the Hirshfeld surface of (I) mapped over (*a*) *d*_norm_, (*b*) inter­molecular C—H⋯O inter­actions and (*c*) shape-index.

**Figure 5:**
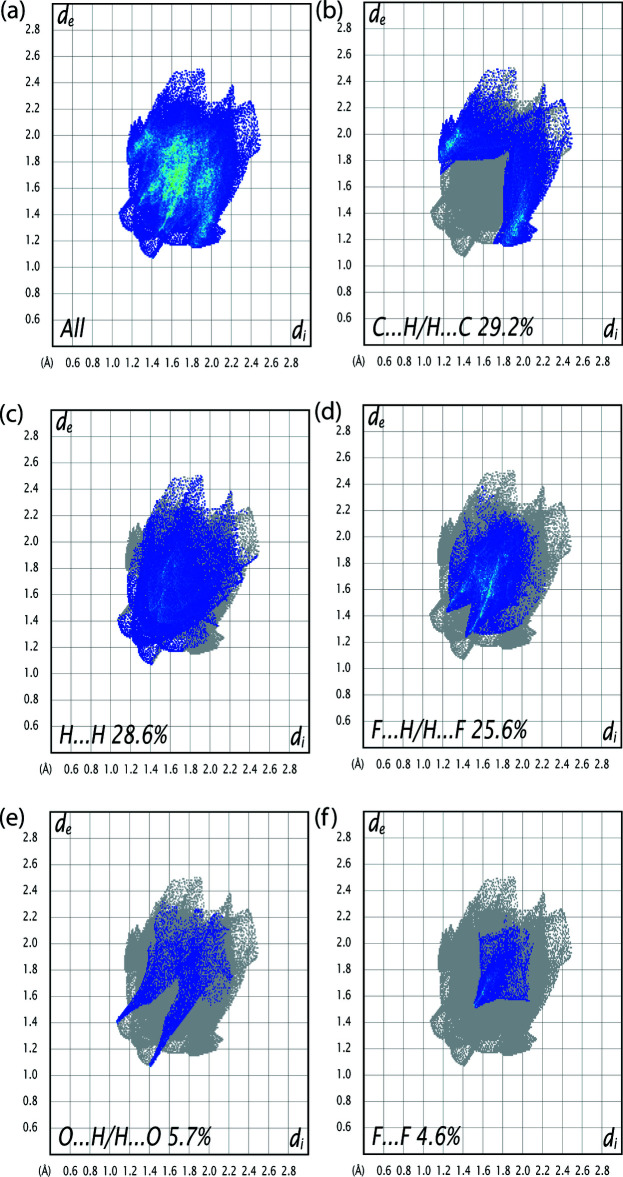
(*a*) The overall two-dimensional finger print plot for (I) and those delineated into: (*b*) C⋯H/H⋯C (29.2%), (*c*) H⋯H (28.6%), (*d*) F⋯H/H⋯F (25.6%), (*e*) O⋯H/H⋯O (5.7%) and (*f*) F⋯F (4.6%) contacts.

In order to visualize the role of weak inter­molecular inter­actions in the crystal, a Hirshfeld surface (HS) analysis (Spackman & Jayatilaka, 2009 ▸) was carried out along with the associated two-dimensional fingerprint plots (McKinnon *et al.*, 2007 ▸) generated using *CrystalExplorer17.5* (Turner *et al.*, 2017 ▸). The three-dimensional *d_norm_* (Fig. 4 ▸ *a*) and shape-index (Fig. 4 ▸ *c*) surfaces of (I) are shown with a standard surface resolution and a fixed colour scale of −0.1805 to 1.0413 a.u. The darkest red spots on the Hirshfeld surface indicate contact points with atoms participating in intra­molecular C—H⋯O (Fig. 4 ▸ *b*) inter­actions that involve C1—H1*A* and the oxygen atom O1 of the phenol group (Table 1 ▸). As illustrated in Fig. 5 ▸ *a*, the corres­ponding fingerprint plots for (I) have characteristic pseudo-symmetrical wings along the *d*_e_ and *d*_i_ diagonal axes. The presence of C—H⋯O inter­actions in the crystal is indicated by the pair of characteristic wings in the fingerprint plot delineated into C⋯H/H⋯C (Fig. 5 ▸ *b*) contacts (29.2% contributions to the Hirshfeld surface). In Fig. 5 ▸ *c*, the widely scattered points in the fingerprint plot are related to H⋯H contacts, which make a contribution of 28.6% to the Hirshfeld surface. There are also F⋯H/H⋯F (25.6%; Fig. 5 ▸ *d*), O⋯H/H⋯O (5.7%; Fig. 5 ▸ *e*) and F⋯F (4.6%; Fig. 5 ▸ *f*) contacts, with smaller contributions from N⋯H/H.·N (2.4%), O⋯C/C⋯O (2.2%), F⋯C/C⋯F (0.8%) and O⋯N/N⋯O (0.2%) contacts.

## DFT calculations   

The optimized structure of (I) in the gas phase was generated theoretically *via* density functional theory (DFT) using the standard B3LYP functional and the 6-311G(d,p) basis-set calculations (Becke, 1993 ▸) as implemented in *GAUSSIAN 09* (Frisch *et al.*, 2009 ▸). The theoretical and experimental results are in good agreement (Table 2 ▸). The C8=N1 bond length is 1.283 (8) Å (experimental) and 1.290 Å (calculated) and the C10—O1 bond length is 1.357 (8) Å (experimental) and 1.342 Å (calculated).

The highest-occupied mol­ecular orbital (HOMO) and the lowest-unoccupied mol­ecular orbital (LUMO) are very important aspects as many electronic, optical and chemical reactivity properties of compounds can be predicted from these frontier mol­ecular orbitals (Tanak, 2019 ▸). A mol­ecule with a small HOMO–LUMO bandgap is more polarizable than one with a large gap and is considered a ‘soft’ mol­ecule because of its high polarizibility while mol­ecules with a large bandgap are considered to be ‘hard’ mol­ecules. To better understand the nature of (I), the electron affinity (*A* = −*E*_HOMO_), the ionization potential (*I* = −*E*_LUMO_), the HOMO–LUMO energy gap (Δ*E*), the chemical hardness (η) and softness (*S*) (based on the *E*_HOMO_ and *E*_LUMO_ energies; Tanak, 2019 ▸) were calculated (Table 3 ▸). Based on the relatively large Δ*E* and η values, the title compound can be classified as a hard mol­ecule.

**Figure 6:**
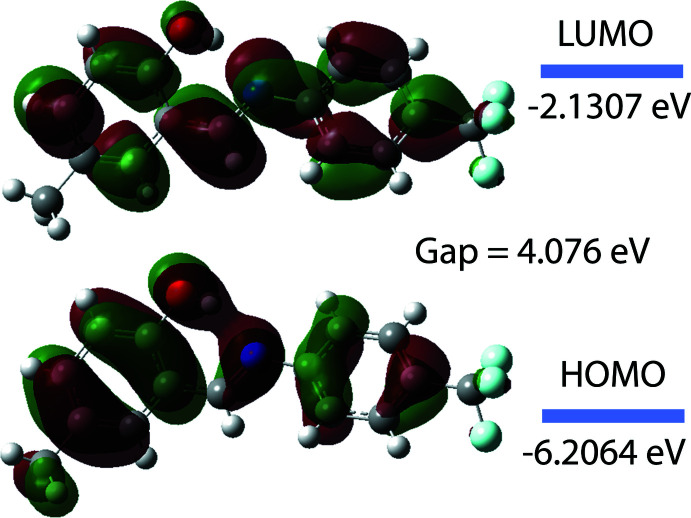
The energy band gap of (I).

The electron distribution of the HOMO and LUMO energy levels is shown in Fig. 6 ▸. The DFT study shows that the HOMO and LUMO are localized in a plane extending over the whole 4-methyl-2-[(4-tri­fluoro­methyl­phenyl­imino)­meth­yl]phenol unit. From the frontier orbital analysis, it can be concluded that a HOMO-to-LUMO excitation of (I) would be a π–π* transition that would weaken the imine bond and drive the production of an excited-state keto–amine tautomer from the enol–imine ground state observed in the solid state. The calculated band gap of (I) is 4.076 eV, which is similar to that reported for other Schiff base materials, such as for example (*E*)-2-{[(3-chloro­phen­yl)imino]­meth­yl}-6-methyl­phenol (energy gap = 4.069 eV; Faizi *et al.*, 2019 ▸) and (*E*)-2-[(2-hy­droxy-5-meth­oxy­benzyl­idene)amino]­benzo­nitrile (energy gap = 3.520 eV; Saraçoğlu *et al.*, 2020 ▸).

## Database survey   

A search of the Cambridge Structural Database (CSD, version 5.40, update of November 2018; Groom *et al.*, 2016 ▸) for the (*Z*)-1-phenyl-*N*-[3-(tri­fluoro­methyl­phen­yl]methanimine skeleton yielded seven matches. Metal complexes with ligands analogous to (I) are the ruthenium complex chloro-(1-methyl-4-(propan-2-yl)benzene)-(2-({[4-(tri­fluoro­meth­yl)phen­yl]im­ino}­meth­yl)phenolato)ruthenium(II) (BIHCED; Cassells *et al.*, 2018 ▸), the rhodium complex (η^5^-penta­methyl­cyclo­penta­dien­yl)chlorido­[2-({[4-(tri­fluoro­meth­yl)phen­yl]imino}­meth­yl)phenolato]rhodium(III) (BIHCIH; Cassells *et al.*, 2018 ▸) and the iridium complex (η^5^-penta­methyl­cyclo­penta­dien­yl)chlorido­[2-({[4-(tri­fluoro­meth­yl) phen­yl]imino}­meth­yl)phen­olato]iridium(III) (BIHCON; Cassells *et al.*, 2018 ▸). Other similar ligands are incorporated into the titanium complex di­chlorido­bis­(3,5-di-*tert*-butyl-*N*-(4-tri­fluoro­methyl­phen­yl)sal­icylaldiminato)titanium(IV) toluene solvate (INOTUA; Mason *et al.*, 2002 ▸) and the copper complex bis­{4-tri­fluoro­methyl­phen­yl[(2-oxo-3*H*-naphth-3-yl­idene)meth­yl]amin­ato}copper(II) (POPFEF; Fernández *et al.*, 1994 ▸). Two vanadium complexes with ligands similar to that in (I) are di­chlorido­{2-[*N*-(4-tri­fluoro­methyl­phen­yl)imino­meth­yl]phenolato}bis(tetra­hydro­furan)­vanadium(III) (YOGSUJ; Wu *et al.*, 2008 ▸) and chlorido­bis­{2-[*N*-(4-tri­fluoro­methyl­phen­yl)imino­meth­yl]phenolato}(tetra­hydro­furan)­vanadium(III) (YOGTOE; Wu *et al.*, 2008 ▸). Similar uncomplexed Schiff base mol­ecules are *N*-[3,5-bis­(tri­fluoro­meth­yl)phen­yl]-3-meth­oxy­salicylaldimine (Karadayı *et al.*, 2015 ▸), 2-{[3,5-bis­(tri­fluoro­meth­yl)phen­yl]carbonoimido­yl}phenol (Yıldız *et al.*, 2015 ▸), 2-{[3,5-bis­(tri­fluoro­meth­yl)phen­yl]carbonoimido­yl}phenol (Ünver *et al.*, 2016 ▸), (*E*)-3-{[3-(tri­fluoro­meth­yl)phenyl­imino]­meth­yl}benz­ene-1,2-diol (Koşar *et al.*, 2010 ▸), 2-fluoro-*N*-(3-nitro­benzyl­idene)-5-(tri­fluoro­meth­yl)aniline (Yang *et al.*, 2007 ▸), (*E*)-2-meth­yl-6-[3-(tri­fluoro­meth­yl)phenyl­imino­meth­yl]phenol (Akkaya *et al.*, 2007 ▸), (*E*)-2-[(4-chloro­phen­yl)imino­meth­yl]-4-(tri­fluoro­meth­oxy)phenol (Tüfekçi *et al.*, 2009 ▸) and (*E*)-4-methyl-2-[3-(tri­fluoro­meth­yl)phenyl­imino­meth­yl]phenol (Gül *et al.*, 2007 ▸). The C=N bond lengths in these structures vary from 1.270 (3) to 1.295 (5) Å and the C—O bond lengths from 1.336 (5) to 1.366 (2) Å. The mol­ecular configurations of these structures are also not planar, with dihedral angles between the phenyl rings varying between 5.00 (5) and 47.62 (9)°. It is likely that the intra­molecular O—H⋯N hydrogen bond, where the imine *N* atom acts as an hydrogen-bond acceptor, is an important prerequisite for the tautomeric shift toward the phenol–imine form. In fact, in all eight structures of the phenol–imine tautomers, hydrogen bonds of this type are observed.

## Synthesis and crystallization   

The title compound was prepared by combining solutions of 2-hy­droxy-5-methyl­benzaldehyde (38.0 mg, 0.28 mmol) in ethanol (15 mL) and 4-tri­fluoro­methyl­phenyl­amine (42.0 mg, 0.28 mmol) in ethanol (15 mL) and stirring the mixture for 8 h under reflux. Single crystals suitable for X-ray analysis were obtained by slow evaporation of an ethanol solution (yield 65%, m.p. 425–427K).

## Refinement   

Crystal data, data collection and structure refinement details are summarized in Table 4 ▸. All C-bound H atoms were positioned geometrically and refined using a riding model with C—H = 0.93–0.97 Å and with *U*_iso_(H) = 1.2–1.5*U*_eq_(C). The hydrogen atom of the phenol group was located in a difference map and also included as a riding contributor with O—H = 0.82 Å and *U*_iso_(H) = 1.5*U*_eq_(O). During refinement, the twin transformation matrix (−1.0 0.0 0.0, 0.0 −1.0 0.0, 0.0 0.0 −1.0), was used.

## Supplementary Material

Crystal structure: contains datablock(s) I. DOI: 10.1107/S2056989020009615/mw2164sup1.cif

Structure factors: contains datablock(s) I. DOI: 10.1107/S2056989020009615/mw2164Isup2.hkl

Supporting information file. DOI: 10.1107/S2056989020009615/mw2164Isup3.cml

CCDC reference: 2016363

Additional supporting information:  crystallographic information; 3D view; checkCIF report

## Figures and Tables

**Table 1:** Hydrogen-bond geometry (Å, °)

*D*—H⋯*A*	*D*—H	H⋯*A*	*D*⋯*A*	*D*—H⋯*A*
O1—H1⋯N1	0.82	1.90	2.620 (7)	146
C1—H1*A*⋯O1^i^	0.93	2.60	3.463 (7)	154

Symmetry code: (i) 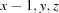.

**Table 2:** Comparison of selected observed (X-ray data) and calculated (DFT) geometric parameters (Å, °) for (I)

Parameter	X-ray	B3LYP/6–311G(d,p)
O1—C10	1.357 (8)	1.342
N1—C8	1.283 (8)	1.290
C3—C7	1.497 (9)	1.502
C6—N1	1.410 (7)	1.404
C8—C9	1.430 (9)	1.446
N1—C8—C9	123.9 (6)	122.6
C8—N1—C6	122.2 (5)	121.0
O1—C10—C9	122.1 (5)	122.3

**Table 3:** Inter­action energies for (I)

Mol­ecular Energy (a.u.) (eV)	Compound (I)
Total Energy *TE* (eV)	−27438.7489
*E*_HOMO_ (eV)	−6.2064
*E*_LUMO_ (eV)	−2.1307
Gap, *ΔE* (eV)	4.076
Dipole moment, *μ* (Debye)	4.466
Ionization potential, *I* (eV)	6.2064
Electron affinity, *A*	2.1307
Electronegativity, *χ*	4.1685
Hardness, *η*	2.038
Electrophilicity index, *ω*	4.2631
Softness, *σ*	0.245
Fraction of electrons transferred, *ΔN*	0.695

**Table 4:** Experimental details

Crystal data	
Chemical formula	C_15_H_12_F_3_NO
*M*_r_	279.26
Crystal system, space group	Orthorhombic, *P* *c* *a*2_1_
Temperature (K)	296
*a*, *b*, *c* (Å)	6.2592 (5), 7.2229 (6), 28.551 (3)
*V* (Å^3^)	1290.77 (19)
*Z*	4
Radiation type	Mo *K*α
μ (mm^−1^)	0.12
Crystal size (mm)	0.72 × 0.55 × 0.22

Data collection	
Diffractometer	Stoe IPDS 2
Absorption correction	Integration (*X-RED32*; Stoe & Cie, 2002 ▸)
*T*_min_, *T*_max_	0.936, 0.982
No. of measured, independent and observed [*I* > 2σ(*I*)] reflections	6209, 2135, 1517
*R*_int_	0.111
(sin θ/λ)_max_ (Å^−1^)	0.622

Refinement	
*R*[*F* ^2^ > 2σ(*F* ^2^)], *wR*(*F* ^2^), *S*	0.073, 0.213, 0.99
No. of reflections	2135
No. of parameters	182
No. of restraints	1
H-atom treatment	H-atom parameters constrained
Δρ_max_, Δρ_min_ (e Å^−3^)	0.24, −0.22
Absolute structure	Refined as an inversion twin

Computer programs: *X-AREA* and *X-RED32* (Stoe & Cie, 2002 ▸), *SHELXT2018/3* (Sheldrick, 2015*a* ▸), *SHELXL2018/3* (Sheldrick, 2015*b* ▸), *OLEX2* (Dolomanov *et al.*, 2009 ▸), *Mercury* (Macrae *et al.*, 2020 ▸), *WinGX* (Farrugia, 2012 ▸), *PLATON* (Spek, 2020 ▸) and *publCIF* (Westrip, 2010 ▸).

## supplementary crystallographic information

### Crystal data

**Table:**

C_15_H_12_F_3_NO	*D*_x_ = 1.437 Mg m^−^^3^
*M**_r_* = 279.26	Mo *K*α radiation, λ = 0.71073 Å
Orthorhombic, *P**c**a*2_1_	Cell parameters from 7251 reflections
*a* = 6.2592 (5) Å	θ = 2.8–26.8°
*b* = 7.2229 (6) Å	µ = 0.12 mm^−^^1^
*c* = 28.551 (3) Å	*T* = 296 K
*V* = 1290.77 (19) Å^3^	Prism, yellow
*Z* = 4	0.72 × 0.55 × 0.22 mm
*F*(000) = 576	

### Data collection

**Table:**

Stoe IPDS 2 diffractometer	2135 independent reflections
Radiation source: sealed X-ray tube, 12 x 0.4 mm long-fine focus	1517 reflections with *I* > 2σ(*I*)
Plane graphite monochromator	*R*_int_ = 0.111
Detector resolution: 6.67 pixels mm^-1^	θ_max_ = 26.2°, θ_min_ = 2.8°
rotation method scans	*h* = −7→6
Absorption correction: integration (X-RED32; Stoe & Cie, 2002)	*k* = −8→8
*T*_min_ = 0.936, *T*_max_ = 0.982	*l* = −28→34
6209 measured reflections	

### Refinement

**Table:**

Refinement on *F*^2^	Secondary atom site location: difference Fourier map
Least-squares matrix: full	Hydrogen site location: inferred from neighbouring sites
*R*[*F*^2^ > 2σ(*F*^2^)] = 0.073	H-atom parameters constrained
*wR*(*F*^2^) = 0.213	*w* = 1/[σ^2^(*F*_o_^2^) + (0.1465*P*)^2^] where *P* = (*F*_o_^2^ + 2*F*_c_^2^)/3
*S* = 0.99	(Δ/σ)_max_ < 0.001
2135 reflections	Δρ_max_ = 0.24 e Å^−^^3^
182 parameters	Δρ_min_ = −0.22 e Å^−^^3^
1 restraint	Absolute structure: Refined as an inversion twin
Primary atom site location: dual	Absolute structure parameter: 0 (3)

### Special details

**Table:**

Geometry. All esds (except the esd in the dihedral angle between two l.s. planes) are estimated using the full covariance matrix. The cell esds are taken into account individually in the estimation of esds in distances, angles and torsion angles; correlations between esds in cell parameters are only used when they are defined by crystal symmetry. An approximate (isotropic) treatment of cell esds is used for estimating esds involving l.s. planes.
Refinement. Refined as a two-component inversion twin

### Fractional atomic coordinates and isotropic or equivalent isotropic displacement parameters (Å^2^)

**Table:**

	*x*	*y*	*z*	*U*_iso_*/*U*_eq_	
N1	−0.2713 (8)	−0.7682 (6)	−0.49830 (18)	0.0645 (11)	
O1	0.0688 (7)	−0.6785 (7)	−0.54674 (17)	0.0809 (12)	
H1	0.000741	−0.705484	−0.523169	0.121*	
F2	−0.8837 (9)	−0.7711 (8)	−0.3228 (2)	0.1216 (18)	
C10	−0.0518 (9)	−0.7125 (6)	−0.5853 (2)	0.0630 (13)	
C6	−0.3839 (8)	−0.7631 (6)	−0.4556 (2)	0.0570 (12)	
F1	−0.6915 (9)	−0.5479 (6)	−0.30622 (18)	0.1272 (19)	
C5	−0.2780 (10)	−0.8240 (7)	−0.4157 (2)	0.0655 (14)	
H5	−0.141666	−0.874119	−0.418207	0.079*	
C3	−0.5758 (10)	−0.7369 (7)	−0.3684 (2)	0.0626 (13)	
C4	−0.3737 (10)	−0.8105 (7)	−0.3725 (2)	0.0657 (14)	
H4	−0.301758	−0.851192	−0.345952	0.079*	
C14	−0.3760 (10)	−0.8129 (7)	−0.6233 (2)	0.0640 (13)	
H14	−0.513415	−0.861146	−0.621131	0.077*	
C7	−0.6847 (12)	−0.7213 (8)	−0.3219 (2)	0.0744 (16)	
C9	−0.2636 (9)	−0.7789 (6)	−0.5821 (2)	0.0628 (13)	
C8	−0.3645 (10)	−0.8001 (7)	−0.5375 (2)	0.0657 (14)	
H8	−0.505930	−0.839357	−0.536897	0.079*	
F3	−0.5971 (13)	−0.8224 (11)	−0.29008 (19)	0.160 (3)	
C12	−0.0825 (12)	−0.7083 (8)	−0.6683 (2)	0.0719 (16)	
H12	−0.020903	−0.683218	−0.697211	0.086*	
C1	−0.5870 (9)	−0.6869 (7)	−0.4518 (2)	0.0638 (13)	
H1A	−0.658405	−0.646274	−0.478455	0.077*	
C11	0.0350 (11)	−0.6758 (7)	−0.6286 (3)	0.0745 (15)	
H11	0.173005	−0.628998	−0.630903	0.089*	
C2	−0.6836 (10)	−0.6716 (7)	−0.40787 (19)	0.0627 (13)	
H2	−0.818272	−0.618523	−0.404942	0.075*	
C13	−0.2928 (12)	−0.7782 (7)	−0.6671 (2)	0.0706 (15)	
C15	−0.4146 (15)	−0.8082 (10)	−0.7111 (3)	0.089 (2)	
H15A	−0.326864	−0.775423	−0.737439	0.134*	
H15B	−0.454828	−0.936105	−0.713450	0.134*	
H15C	−0.540560	−0.732414	−0.710949	0.134*	

### Atomic displacement parameters (Å^2^)

**Table:**

	*U*^11^	*U*^22^	*U*^33^	*U*^12^	*U*^13^	*U*^23^
N1	0.069 (3)	0.072 (2)	0.053 (3)	−0.0016 (18)	0.003 (2)	0.002 (2)
O1	0.067 (3)	0.109 (3)	0.067 (3)	−0.016 (2)	−0.006 (2)	−0.002 (2)
F2	0.110 (3)	0.159 (4)	0.096 (3)	−0.050 (3)	0.031 (3)	−0.024 (3)
C10	0.058 (3)	0.065 (3)	0.066 (3)	0.001 (2)	−0.002 (3)	−0.001 (2)
C6	0.058 (3)	0.056 (2)	0.057 (3)	−0.0059 (19)	0.004 (2)	0.003 (2)
F1	0.176 (5)	0.098 (3)	0.108 (3)	−0.023 (3)	0.049 (4)	−0.037 (2)
C5	0.068 (3)	0.068 (3)	0.060 (3)	0.006 (2)	−0.001 (3)	−0.002 (2)
C3	0.070 (4)	0.062 (3)	0.056 (3)	−0.002 (2)	0.003 (3)	0.006 (2)
C4	0.074 (4)	0.065 (3)	0.059 (3)	0.001 (2)	−0.006 (3)	0.009 (2)
C14	0.063 (3)	0.071 (2)	0.059 (3)	0.000 (2)	0.004 (3)	−0.005 (2)
C7	0.092 (4)	0.073 (3)	0.058 (3)	−0.003 (3)	0.000 (3)	−0.003 (3)
C9	0.065 (3)	0.059 (2)	0.064 (3)	0.002 (2)	0.005 (3)	0.005 (2)
C8	0.065 (4)	0.067 (3)	0.064 (4)	−0.005 (2)	0.005 (3)	0.002 (2)
F3	0.200 (7)	0.207 (6)	0.072 (3)	0.102 (5)	0.042 (3)	0.052 (3)
C12	0.089 (5)	0.069 (3)	0.058 (3)	0.003 (3)	0.007 (3)	0.000 (2)
C1	0.066 (3)	0.072 (3)	0.054 (3)	−0.001 (2)	−0.008 (2)	0.005 (2)
C11	0.074 (4)	0.072 (3)	0.078 (4)	−0.001 (2)	0.006 (3)	0.002 (3)
C2	0.060 (3)	0.069 (3)	0.060 (3)	0.003 (2)	−0.006 (3)	0.001 (2)
C13	0.085 (4)	0.067 (3)	0.060 (3)	0.001 (3)	−0.002 (3)	0.000 (2)
C15	0.113 (6)	0.095 (4)	0.060 (4)	−0.007 (4)	−0.011 (3)	−0.002 (3)

### Geometric parameters (Å, º)

**Table:**

N1—C8	1.283 (8)	C14—C9	1.391 (9)
N1—C6	1.410 (7)	C14—H14	0.9300
O1—C10	1.357 (8)	C7—F3	1.289 (8)
O1—H1	0.8200	C9—C8	1.430 (9)
F2—C7	1.297 (9)	C8—H8	0.9300
C10—C11	1.376 (10)	C12—C11	1.371 (10)
C10—C9	1.412 (8)	C12—C13	1.410 (10)
C6—C5	1.389 (8)	C12—H12	0.9300
C6—C1	1.389 (8)	C1—C2	1.397 (8)
F1—C7	1.331 (7)	C1—H1A	0.9300
C5—C4	1.374 (8)	C11—H11	0.9300
C5—H5	0.9300	C2—H2	0.9300
C3—C4	1.377 (9)	C13—C15	1.487 (10)
C3—C2	1.395 (8)	C15—H15A	0.9600
C3—C7	1.497 (9)	C15—H15B	0.9600
C4—H4	0.9300	C15—H15C	0.9600
C14—C13	1.377 (9)		
			
C8—N1—C6	122.2 (5)	C14—C9—C8	120.7 (5)
C10—O1—H1	109.5	C10—C9—C8	120.5 (6)
O1—C10—C11	118.3 (5)	N1—C8—C9	123.9 (6)
O1—C10—C9	122.2 (6)	N1—C8—H8	118.0
C11—C10—C9	119.5 (6)	C9—C8—H8	118.0
C5—C6—C1	119.9 (5)	C11—C12—C13	122.8 (6)
C5—C6—N1	117.6 (5)	C11—C12—H12	118.6
C1—C6—N1	122.3 (5)	C13—C12—H12	118.6
C4—C5—C6	120.3 (5)	C6—C1—C2	119.8 (5)
C4—C5—H5	119.8	C6—C1—H1A	120.1
C6—C5—H5	119.8	C2—C1—H1A	120.1
C4—C3—C2	120.4 (6)	C12—C11—C10	119.8 (6)
C4—C3—C7	121.5 (6)	C12—C11—H11	120.1
C2—C3—C7	118.1 (6)	C10—C11—H11	120.1
C5—C4—C3	120.3 (6)	C3—C2—C1	119.3 (5)
C5—C4—H4	119.9	C3—C2—H2	120.4
C3—C4—H4	119.9	C1—C2—H2	120.4
C13—C14—C9	122.9 (6)	C14—C13—C12	116.1 (6)
C13—C14—H14	118.6	C14—C13—C15	123.2 (7)
C9—C14—H14	118.6	C12—C13—C15	120.7 (6)
F3—C7—F2	105.4 (7)	C13—C15—H15A	109.5
F3—C7—F1	108.0 (7)	C13—C15—H15B	109.5
F2—C7—F1	103.7 (6)	H15A—C15—H15B	109.5
F3—C7—C3	112.9 (6)	C13—C15—H15C	109.5
F2—C7—C3	113.5 (5)	H15A—C15—H15C	109.5
F1—C7—C3	112.6 (5)	H15B—C15—H15C	109.5
C14—C9—C10	118.8 (5)		
			
C8—N1—C6—C5	147.0 (5)	O1—C10—C9—C8	4.5 (7)
C8—N1—C6—C1	−38.2 (7)	C11—C10—C9—C8	−173.6 (5)
C1—C6—C5—C4	0.5 (7)	C6—N1—C8—C9	170.1 (4)
N1—C6—C5—C4	175.5 (4)	C14—C9—C8—N1	−178.8 (5)
C6—C5—C4—C3	0.2 (8)	C10—C9—C8—N1	−2.6 (8)
C2—C3—C4—C5	−1.4 (8)	C5—C6—C1—C2	0.1 (7)
C7—C3—C4—C5	179.7 (5)	N1—C6—C1—C2	−174.7 (5)
C4—C3—C7—F3	−16.4 (9)	C13—C12—C11—C10	0.3 (8)
C2—C3—C7—F3	164.6 (7)	O1—C10—C11—C12	−179.9 (5)
C4—C3—C7—F2	−136.3 (6)	C9—C10—C11—C12	−1.7 (8)
C2—C3—C7—F2	44.7 (7)	C4—C3—C2—C1	2.0 (8)
C4—C3—C7—F1	106.3 (7)	C7—C3—C2—C1	−179.1 (5)
C2—C3—C7—F1	−72.7 (7)	C6—C1—C2—C3	−1.3 (8)
C13—C14—C9—C10	−2.5 (7)	C9—C14—C13—C12	1.1 (8)
C13—C14—C9—C8	173.8 (5)	C9—C14—C13—C15	−177.9 (5)
O1—C10—C9—C14	−179.1 (5)	C11—C12—C13—C14	0.0 (8)
C11—C10—C9—C14	2.7 (7)	C11—C12—C13—C15	179.0 (6)

### Hydrogen-bond geometry (Å, º)

**Table:**

*D*—H···*A*	*D*—H	H···*A*	*D*···*A*	*D*—H···*A*
O1—H1···N1	0.82	1.90	2.620 (7)	146
C1—H1*A*···O1^i^	0.93	2.60	3.463 (7)	154

Symmetry code: (i) *x*−1, *y*, *z*.
